# Efficacy of vision restoration therapy after optic neuritis (VISION study): study protocol for a randomized controlled trial

**DOI:** 10.1186/1745-6215-13-94

**Published:** 2012-06-28

**Authors:** Johann Schinzel, Lina Schwarzlose, Holger Dietze, Karolina Bartusch, Susanne Weiss, Stephanie Ohlraun, Friedemann Paul, Jan Dörr

**Affiliations:** 1 NeuroCure Clinical Research Center, Charité - Universitaetsmedizin Berlin, Berlin, Germany; 2 Beuth University of Applied Sciences, Berlin, Germany; 3 Clinical and Experimental Multiple Sclerosis Research Center, Charité - Universitaetsmedizin Berlin, Berlin, Germany; 4 Experimental and Clinical Research Center, Max Delbrueck Center for Molecular Medicine, Charité - Universitaetsmedizin Berlin, Berlin, Germany

**Keywords:** Multiple sclerosis, Neuroplasticity, Optic neuritis, Treatment, Vision restoration therapy, Visual function

## Abstract

**Background:**

Optic neuritis is a frequent manifestation of multiple sclerosis. Visual deficits range from a minor impairment of visual functions through to complete loss of vision. Although many patients recover almost completely, roughly 35% of patients remain visually impaired for years, and therapeutic options for those patients hardly exist. Vision restoration therapy is a software-based visual training program that has been shown to improve visual deficits after pre- and postchiasmatic injury. The aim of this pilot study is to evaluate whether residual visual deficits after past or recent optic neuritis can be reduced by means of vision restoration therapy.

**Methods/design:**

A randomized, controlled, patient- and observer-blinded clinical pilot study (VISION study) was designed to evaluate the efficacy of vision restoration therapy in optic neuritis patients. Eighty patients with a residual visual deficit after optic neuritis (visual acuity ≤0.7 and/or scotoma) will be stratified according to the time of optic neuritis onset (manifestation more than 12 months ago (40 patients, fixed deficit) versus manifestation 2 to 6 months ago (40 patients, recent optic neuritis)), and randomized into vision restoration therapy arm or saccadic training arm (control intervention). Patients will be instructed to complete a computer-based visual training for approximately 30 minutes each day for a period of 6 months. Patients and evaluators remain blinded to the treatment allocation throughout the study. All endpoints will be analyzed and *P*-values < 0.05 will be considered statistically significant. The primary outcome parameter will be the expansion of the visual field after 3 and 6 months of treatment as determined by static visual field perimetry and high resolution perimetry. Secondary outcome variables will include visual acuity at both low and high contrast, glare contrast sensitivity, visually evoked potentials, optical coherence tomography and other functional tests of the visual system, alertness, health-related quality of life, fatigue, and depression.

**Discussion:**

If vision restoration therapy is shown to improve visual function after optic neuritis, this method might be a first therapeutic option for patients with incomplete recovery from optic neuritis.

**Trial registration:**

NCT01274702

## Background

Multiple sclerosis (MS) is one of the most frequent chronic diseases of the central nervous system (CNS) in young adults in western countries. The underlying pathomechanism is considered to be an autoimmune mediated attack against CNS structures that leads to CNS demyelination and neuroaxonal degeneration, and ultimately results in CNS dysfunction [1]. The course of the disease is typically relapsing-remitting in the beginning, followed by a secondary chronically progressive phase that is characterized by the accumulation of persistent neurological deficits. The therapeutic options comprise high-dose glucocorticosteroids in the acute phase and long-term immunomodulation or -suppression depending on the course and the dynamics [2]. An optic neuritis (ON) is the first symptom in 20 to 30% of MS patients, and approximately 50% of MS patients develop an ON at some point [3-6]. Leading symptoms of ON are reduced visual acuity, periocular pain (especially during eye movements), reduced contrast sensitivity, dysfunction of color vision, and visual field defects. In the majority of cases the loss of vision develops subacutely over a few days, whereas the recovery of the visual functions usually takes some weeks [3]. Although many patients recover almost completely, roughly 35% of patients remain visually impaired even after 10 years of onset [7,8]. Importantly, incomplete recovery of vision often leads to a reduced quality of life [9], and therapeutic options for those patients hardly exist [10].

Previous studies suggest beneficial effects of neuroplastic approaches for the treatment of visual deficits of neurological etiology [8,9]. Based on experiments with repetitive measurements of incremental thresholds at the edges of absolute visual field defects, Kasten and colleagues developed a software-based training program which is referred to as “vision restoration therapy” (VRT) [11]. The program (NovaVision AG, Magdeburg, Germany) can be run on a standard personal computer, which makes it suitable for home-based training. In a randomized, placebo-controlled, double-blind study on patients with pre- and postchiasmatic injury, VRT treatment expanded the mean visual field in the postchiasmatic group by 4.9° (± 1.7) whereas the mean visual field of the placebo group was reduced by 0.9° (± 0.8). The subgroup of patients with optic nerve injuries showed a trend towards a visual field expansion of 5.8° (± 1.2) in the VRT group compared to 4.3° (± 0.7) in the placebo group [11]. In patients with anterior ischemic optic neuropathy (AION), VRT resulted in a significant improvement of binocular reading speed and revealed positive trends on improvement of visual acuity, foveal sensitivity, and high-resolution perimetry (HRP). Moreover, all patients reported a subjective improvement of their visual function [12]. VRT is based on the hypothesis that visual units which are excluded from visual processing can be reintegrated by specific visual stimulation. Because of the diffuse nature of optic nerve lesions it is assumed that a smooth transition exists between severely and only moderately damaged retinal ganglion cells. Anope areas might, therefore, contain a number of capable but inhibited neurons. In fact, it has been demonstrated that even cortical blind patients have a residual visual sensitivity [13], and peripheral visual stimulation induced neural activity of central retinal ganglion cells in eyes with age-related macular degeneration [14]. It therefore appears likely that VRT stimulates residual retinal ganglion cells in visually impaired post-ON patients, and thus improves visual function [15-21].

Against this background, we introduce the protocol for a randomized, controlled clinical pilot study evaluating the efficacy of VRT in patients with incomplete visual recovery upon ON. We hypothesize that VRT is superior to saccadic training which is used here as a control intervention, with respect to i) restitution of residual visual field defects; ii) additional parameters of both visual function and integrity of the visual system including low contrast and color vision, conductivity, and retinal morphology; and iii) improvement in quality of life estimates.

## Methods/design

### Design and setting

The VISION study is a monocentric, randomized, controlled, and double-blind clinical trial conducted at the NeuroCure Clinical Research Center at the Charité - Universitaetsmedizin, Berlin, Germany. Recruitment started in 2011. Eighty patients with a diagnosis of past or recent ON will be stratified according to the onset of ON. The first group will comprise patients with ON manifestation at least 1 year in the past. The second group will comprise patients with ON onset between 2 and 6 months in the past. These two groups will be randomized 1:1 into the VRT group and the control intervention group, with the latter receiving a saccade training (RehaCom, Hasomed GmbH, Magdeburg, Germany). The detailed study design is provided below according to the revised CONSORT statement [22,23].

### Study cohort

Inclusion criteria for participation are: age between 18 and 55 years, ability to provide written informed consent, definite history of ON (supported by objective criteria-like visual evoked potentials (VEP) and/or typical clinical presentation), visual acuity between 0.05 (20/400) and 0.7 (~20/29) or >0.7 and scotoma (a basic visual function must be present to perform the training), and no systemic steroid treatment or plasmapheresis within the last 60 days. Exclusion criteria comprise occurrence of ON ≤ 60 days to avoid confounding by spontaneous resolving optic nerve edema, any other potentially confounding eye diseases (for example, macular degeneration, cataract, glaucoma, diabetic retinopathy), nystagmus, strabismus and amblyopia to avoid fixation errors and impaired binocular function, and a history of epileptic seizures because of repeated presentation of bright light stimuli.

### Randomization and blinding

A freeware program (http://www.random.org) is used to randomize subjects into VRT and control groups. Randomization is performed by an independent person otherwise not involved in the study. Access to the randomization lists is restricted to this person ensuring that the treatment allocation remains concealed to the recruiting and enrolling study personnel. Two members of the study team remain unblinded (KB, SW) in order to instruct and guide patients in all treatment-specific software issues. All information that could possibly reveal the study arm to both patients and blinded evaluators, such as the brand names or labels of the software or user manuals, are carefully avoided. All outcome parameters are determined in a blinded fashion. Patients asking for disclosure of treatment allocation will be put off until the end of the study.

### Ethics

The VISION study has been approved by the local ethics committee (Charité Campus Mitte; Berlin/Germany; no. EA1/078/10) and the Data Protection Department of the Charité and is registered at Clinicaltrials.gov (NCT01274702). The study will be conducted in accordance with the Declaration of Helsinki in its current version, the guidelines of the International Conference on Harmonization of Good Clinical Practice (ICH-GCP), and the applicable German laws. All participants are required to give informed written consent. No patient will be deprived of receiving a standard therapy.

### Treatment

#### Vision restoration therapy

VRT is a commercially distributed computer-based visual training program (NovaVison AG, Magdeburg, Germany). This software is based on the measurement of the visual field at baseline using HRP that detects the horizontal 43 degrees and vertical 32 degrees of the visual field like a common perimeter with supra-threshold stimuli and a higher resolution. The aim is to determine the border between a scotoma and normal areas of the visual field in a more precise way than standard automated perimetry does. The VRT software uses algorithms based on the HRP data to stimulate specifically receptive fields close to scotoma borders. VRT is performed using a chin support placed in front of the monitor at a distance according to the size of the monitor to ensure that the light beam of the stimuli reaches the correct areas of the retina. Patients are instructed to exercise for 30 minutes every day over a total period of 6 months. During each training session, 400 white light supra-threshold stimuli are presented on a computer-screen (duration 2000 ms each) and have to be confirmed by the patients by pressing a key within 1000 ms. Central fixation is important and is facilitated by a central color-changing dot (31 pixels) on which the patient continuously has to fixate and confirm each color change by pressing a key. For a maximum training effect, stimulus parameters such as the areas of stimulus presentation, stimulus size, duration and brightness undergo monthly adaptation to any change in the visual field.

#### Control intervention

The control treatment is a computer-based training for rapid eye movements and is part of a comprehensive cognitive rehabilitation/brain performance training software suite (RehaCom, Hasomed GmbH, Magdeburg, Germany) [24]. The patient is asked fixate a central target and to respond to peripheral stimuli moving towards the center of the visual field. As in the VRT arm, a chin support is provided. Some guidance is given, for example a horizontal line that facilitates the differentiation between the fixation target and the moving object. The program has ascending levels of difficulty depending on the size, the contrast, and the velocity of the moving object. As in the VRT arm, patients are instructed to exercise for 30 minutes each day, for 6 months in total. The control treatment enables the patient to compensate loss of visual function by the use of saccadic eye movements [25] rather than inducing a true recovery of visual dysfunction.

### Outcome parameters

The primary outcome parameter is the extend of the visual field after 3 and 6 months of training as determined by both HRP (see above) and threshold 30 degree automated perimetry (HFA 720, Zeiss Meditec AG, Germany). The secondary outcome parameters are outlined below and will be evaluated as displayed in Table 1. Visual acuity measurements are made using the Freiburg Visual Acuity and Contrast Test (FRACT): Landolt rings (ISO 8596) with a constant high contrast and decreasing size are presented at 4 m distance on a computer screen, and the patient is asked to report the perceived direction of the opening of each Landolt ring presented [26]. Contrast sensitivity is measured using FRACT with Landolt rings of equal size but decreasing contrast. Measurements of glare contrast sensitivity are made using the FRACT program as described above and a purpose built glare source (Beuth University of Applied Sciences, Berlin, Germany). The glare source consists of a white frame with eight white LEDs (approximately 8,500 LUX each) circularly arranged around the TFT-screen used for FRACT testing. Recording of VEP allows the conductance velocity and the amplitude of the visual signal evoked by stimulating the retina to be measured. In ON, both a delay of latency and lowered amplitude of this signal is typical. Spectral domain optical coherence tomography (OCT; Spectralis, Heidelberg Engineering, Germany; software version 5.1) is used to measure the retinal nerve fiber layer thickness (RNFLT) of the retina circularly around the optic nerve head and the volume of the macula, as both parameters are increasingly recognized as markers for neuroaxonal degeneration in MS [27]. Color perception is tested using pseudo-iso-chromatic plates (Ishihara, 14-plate version) and a Multi Color Anomaloscope (HMC, Oculus GmbH, Wetzlar, Germany), whereby the Ishihara test serves as a screening-test which is followed by an anomaloscope measurement in case of any abnormality [28]. The quality of stereoscopic vision is assessed using the stereo fly test (Titmus test). The patient wears polarized glasses and views a polarized fly. In normal stereoscopic vision a three-dimensional image of the fly is perceived. The cover/uncover test is performed to detect any strabismus or heterophoria. The International Reading Speed Texts (IReST) are used to measure the reading ability [29]. The Trail Making Test (TMT) is a popular neuropsychological test that provides information on visual search, scanning, speed of processing, mental flexibility, and executive functions. The patient is asked to connect 25 randomly spread numbers on a sheet of paper, and the time required for this task is measured [30]. The Tests of Attentional Performance (TAP 1.7) measure alertness via reaction time and provide a global measure of processing speed of the brain [31]. Alertness is defined as a state of general wakefulness in which a person can quickly react to concrete requirements. The questionnaires that are used to evaluate the health-related quality of life (HRQL) with regard to vision are the National Eye Institute Visual Functioning Questionnaire (NEI-VFQ 39) [32], the Impact of Visual Impairment Scale (IVIS) (which is the visual subscale of the Multiple Sclerosis Questionnaire of Life Inventory (MSQLI)) and a Visual Analogue Scale (VAS). The Short Form 36 (SF-36) is a generic measurement of the HRQL [33,34], the Health 49 is a freely available questionnaire (http://www.hamburger-module.de) [35] and the Fatigue Severity Scale (FSS) is a self-rating, nine-item questionnaire which measures the severity of fatigue [36] which has been validated in a large sample of MS patients [37]. The Beck Depression Inventory (BDI) is used mainly to control for depression as a possible confounder [38]. Finally, the following demographic data will be collected: disease course (clinically isolated syndrome or relapsing-remitting MS or secondary-progressive MS [39], date of first manifestation, date of first diagnosis, side of ON, current treatment, expanded disability status scale [40]), and vital parameters (blood pressure, heart rate, weight, body height, age, and ethnicity).

**Table 1:** Visits and measurements

**Measurements**	**Visit 0 (baseline visit)**	**Visit 1 (after 3 months)**	**Visit 2 (after 6 months)**
Neurostatus	X		
Visual field	X	X	X
Contrast sensitivity	X	X	X
Glare sensitivity	X	X	X
Visual acuity	X	X	X
Visual evoked potentials	X	X	X
Optical coherence tomography	X	X	X
Color perception	X	X	X
Binocular vision	X	X	X
Eye movement/	X	X	X
Leading eye reading speed	X	X	X
Trial making test	X	X	X
Alertness	X	X	X
Health related quality of life	X	X	X

### Statistical analysis

This study is designed as a pilot study because sufficient data that would allow a reasonable *a priori* calculation of sample size are not yet available. All endpoints will be analyzed and described descriptively. The null hypothesis is as follows: VRT has no influence on the visual outcome after ON. Evaluation of endpoints will be carried out by both “intention-to-treat” and “per-protocol” analyses. Statistical tests and presentations will be appropriate to the category and distribution of the respective variables. All tests will be accomplished with a type one error (α = 0.05; bilateral). All calculations will be performed using SPSS, Version 20 (SPSS, Inc., Chicago, IL, USA).

## Discussion

ON is among the most common manifestations of MS and, although the prognosis is usually quite good, a substantial number of patients retain some degree of visual dysfunction which often has a negative impact on their quality of life [3]. Currently, there are no established therapeutic options for a residual visual deficit after ON. This study aims to evaluate whether VRT is effective in patients suffering from visual impairment after ON.

Some mechanisms have been discussed to explain the functionality of the VRT stimulations: neuroplasticity leading to an optimization of the synaptic connectivity and cortical changes of the structure, activation of partially damaged visual units, an increase in the velocity of the visual information processing, and an increase in the general visual attention [11,12,41]. However, since the efficacy of VRT for other neurological disorders has been repeatedly questioned [42] some important issues need to be addressed. For example, sufficient control of central fixation is crucial to rule out any eye movements during training sessions. Furthermore, the training effect needs to be robust enough to be of clinical relevance and should be detectable not only by HRP. Finally, a placebo effect needs to be sufficiently ruled out. Most of these points have been addressed in a study that confirmed the efficacy of VRT in 302 patients with visual deficits due to cerebral ischemia, hemorrhage, head trauma, tumor removal, and anterior ischemic optic neuropathy [41]. The treatment response was independent of eye movement and could be confirmed by conventional perimetry. Although the study protocol of the VISION study addresses these issues, some important limitations need to be considered. First, given the rather small sample size of 40 patients in each group with past and recent ON (20 patients per treatment arm) the study might be underpowered to detect small treatment effects. On the other hand, in the previous study by Kasten and collegues [11], a treatment effect could be detected in an even smaller cohort. Moreover, since this is the first study that addresses a potential therapeutic effect of VRT in patients with incompletely recovered ON, the intention is rather to corroborate the hypothesis, which then clearly needs to be confirmed in a larger study with a statistically sound sample-size calculation. Second, one might argue that the saccadic training performed by the patients in the control arm is not an appropriate control intervention since one might suspect both an intrinsic therapeutic effect on some visual parameters and an unintentional unblinding. However, creating a real training placebo is methodologically difficult because it is likely that any stimulation may result in an activation of the neuronal network [12]. We chose the saccadic training because mechanisms other than neuroplasticity are considered as being crucial for a potential treatment effect. In saccadic training the patient learns to compensate the lost function by the use of saccadic movements of the eye. It is believed that patients disregard anope areas so that an increase of the amplitude of saccadic movements would help them to extend the affected search field [43]. Thus, saccadic training would not result in a real resolution of a visual field defect. Furthermore, despite major efforts, the maintenance of patient blinding remains challenging since the procedures of VRT and control intervention are not exactly identical. Finally, vision in general is a complex process that is dependent on many factors (for example, daylight, fatigue, attention, etc.) that cannot be taken into consideration due to practicability and other unknown factors.

In conclusion, VRT has the potential to be the first effective restorative treatment option for patients suffering from persistent visual deficits after ON. The intention of the VISION study is to corroborate this hypothesis before the initiation of larger, confirmatory trials.

### Trial status

The VISION study is currently recruiting patients. Sixteen patients have already been randomized, six of them have completed the 6 months of treatment.

## Abbreviations

AION, Anterior ischemic optic neuropathy; BDI, Beck Depression Inventory; CIS, Clinically isolated syndrome; CNS, Central nervous system; FRACT, Freiburg Visual Acuity and Contrast Test; FSS, Fatigue Severity Scale; HRP, High-resolution perimetry; HRQL, Health-related quality of life; IReST, International Reading Speed Texts; IVIS, Impact of Visual Impairment Scale; MS, Multiple sclerosis; MSQLI, Multiple Sclerosis Questionnaire of Life Inventory; NEI-VFQ, National Eye Institute Visual Functioning Questionnaire; OCT, Optical coherence tomography; ON, Optic neuritis; RNFLT, Retinal nerve fiber layer thickness; SF-36, Short Form 36; TAP, Tests of Attentional Performance; TMT, Trail Making Test; VAS, Visual Analogue Scale; VEP, Visual evoked potentials; VRT, Vision restoration therapy.

## Competing interests

This project received support in the form of technical devices, software and helpful knowledge from NovaVision and Hasomed in Magdeburg, Germany. Both companies are neither involved in the study design nor the interpretation of data or publication of results.

## Authors’ contributions

JS: conceptualization and conductance of the study, recruitment and evaluation of patients, drafting of the manuscript. LS: recruitment and evaluation of patients, critical revision of the manuscript. HD: conceptualization of the study, provision of expert knowledge, critical revision of the manuscript. KB: instruction and guidance of patients, critical revision of the manuscript. SW: instruction and guidance of patients, critical revision of the manuscript. SO: conceptualization and administration of the study, critical revision of the manuscript. FP: conceptualization, conductance and supervision of the study, critical revision of the manuscript. JD: conceptualization, conductance and supervision of the study, drafting of the manuscript. All authors read and approved the final manuscript.
